# Particle Diffusometry: An Optical Detection Method for *Vibrio cholerae* Presence in Environmental Water Samples

**DOI:** 10.1038/s41598-018-38056-7

**Published:** 2019-02-11

**Authors:** Katherine N. Clayton, Taylor J. Moehling, Dong Hoon Lee, Steven T. Wereley, Jacqueline C. Linnes, Tamara L. Kinzer-Ursem

**Affiliations:** 1 School of Mechanical Engineering, Purdue University, West Lafayette, IN 47907 USA; 2 Weldon School of Biomedical Engineering, Purdue University, West Lafayette, IN 47907 USA

## Abstract

There is a need for a rapid, robust, and sensitive biosensor to identify low concentrations of pathogens in their native sample matrix without enrichment or purification. Nucleic acid-based detection methods are widely accepted as the gold standard in diagnostics, but robust detection of low concentrations of pathogens remains challenging. Amplified nucleic acids produce more viscous solutions, which can be measured by combining these products with fluorescent particles and measuring the change in the particle diffusion coefficient using a technique known as particle diffusometry. Here, we utilize *Vibrio cholerae (V*. *cholerae)* as a proof-of-concept for our detection system due to its inherently low concentration in environmental water samples. We demonstrate that particle diffusometry can be used to detect down to 1 *V*. *cholerae* cell in molecular-grade water in 20 minutes and 10 *V*. *cholerae* cells in pond water in just 35 minutes in 25 µL reaction volumes. The detection limit in pond water is environmentally relevant and does not require any enrichment or sample preparation steps. Particle diffusometry is 10-fold more sensitive than current gold standard fluorescence detection of nucleic acid amplification. Therefore, this novel measurement technique is a promising approach to detect low levels of pathogens in their native environments.

## Introduction

Environmental pathogen detection presents unique challenges in the development of novel biosensors due to the exceedingly low concentrations of pathogens in their native environments. For example, despite surviving at only 100 cells/mL in environmental water sources, the *Vibrio cholerae (V*. *cholerae)* bacteria that causes the devastating diarrheal disease cholera, leads to over 150,000 deaths worldwide each year^1,2^. Further, the current gold standard for the detection of *V*. *cholerae* in water sources is an 8-hour process involving bacteria enrichment and culture followed by polymerase chain reaction (PCR)^3^. Despite being one of the most sensitive laboratory detection methods, PCR is still not sensitive or robust enough to directly detect *V*. *cholerae* from the environment^1^. Hence, there is a need for a biosensor that can rapidly detect pathogens, such as *V*. *cholerae*, in their native environments.

Next generation mechanical, electrical, or optical signal transducers for biosensing have the potential to detect pathogens and biomolecular species with high sensitivity. For example, mechanical micro and nano-cantilever systems have been used extensively to detect *E*. *coli* in the range of 1–100 cells/mL^4–8^ and *B*. *anthracis* at less than 300 cells/mL^4,9–11^. Additionally, electrical and electrochemical transducers, such as impedance-based sensing of carbon nanotubes where the signal change is caused by *E*. *coli* binding to the surface, have been shown to have a limit of detection (LOD) of 50 colony forming units (cfu)/mL^12^. Further, optical and spectroscopic-based biosensing techniques have been used to achieve highly sensitive detection of pathogens in complex samples such as mixed cultures or food matrices. As few as 10 cfu/mL of *E*. *coli*, *S*. *typhimurium*, and *S*. *aureus* have been detected using surface enhanced Raman spectroscopy (SERS)^13,14^. Dark-field microscopy techniques that detect light scattered from nanoparticles also show promise in pathogen detection applications. For instance, gold nanoparticles were functionalized with antibodies against *E*. *coli* surface antigens and imaged. A color and shape analysis algorithm was applied to the *E*. *coli* dark-field images to detect as little as 10^4^ cfu/mL of bacteria in only 30 minutes^15^. As promising as these technologies are, no single technique overcomes all of the challenges incurred in pathogen identification. In particular, these methods require extensive pre-processing techniques to purify or label samples prior to detection. Indeed, designing an integrated biosensor that rapidly detects pathogens at a low limit of detection in the presence of complex sample matrices continues to be a primary goal in biosensor development^16–18^.

Due to their exquisite sensitivity, nucleic acid amplification methods, such as PCR and loop-mediated isothermal amplification (LAMP), provide excellent target DNA enrichment for biosensor detection. LAMP is a particularly attractive DNA amplification method because it operates at a single temperature and provides rapid and robust amplification even in the presence of complex sample matrices^19^. Amplicon detection has been integrated into LAMP assays using fluorescent, visual, and electrochemical methods^20–22^. Okada *et al*. showed that visual detection of LAMP assays are robust enough to identify *V*. *cholerae* found in clinical rectal swabs^23^. The promising results from Okada *et al*. indicate that LAMP can be used for the detection of the low concentrations of *V*. *cholerae* in complex sample matrices. Indeed, we wish to employ LAMP for the development of an environmental-based biosensor for ultrasensitive detection of *V*. *cholerae* in water samples. Taking advantage of the primer design from Okada *et al*., we have developed a LAMP protocol for *V*. *cholerae* that is efficient (under 30-minute amplification), specific (targeting 6 different regions of the cholera toxin gene), and robust (usable in non-pretreated environmental water sources).

**Figure 1:**
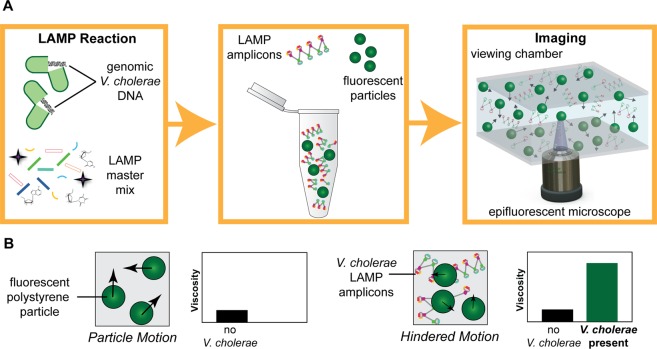
Illustration of PD-LAMP set-up. (**A**) The LAMP assay is performed in the presence of *V*. *cholerae* DNA (left). LAMP amplicons combined with polystyrene fluorescent particles (middle) are imaged under fluorescence microscopy (right). (**B**) Relationship of particle motion and viscosity. Particles undergo Brownian motion in a solution (left). In the presence of LAMP amplicons, the viscosity of the solution increases and particles experience hindered motion, indicating the presence of *V*. *cholerae* DNA in the sample (right).

In this work, we develop a highly accurate and sensitive biosensor for the rapid detection of *V*. *cholerae* in environmental water sources by pairing LAMP with particle diffusometry (PD) (Fig. 1A). PD involves rapid optical measurements of particle Brownian motion^24–26^ following amplification. When *V*. *cholerae* DNA is present in the solution, the LAMP assay polymerizes DNA targets into a variety of base pair lengths up to 25 kilobases^27^. This polymerization causes the particle Brownian motion to decrease^25^. We can calculate this change in the particle Brownian motion with PD using correlation-based algorithms of the particle images^24^ (Fig. 1B). In this work, we show the applicability of PD-LAMP to detect the presence of *V*. *cholerae*. We can use PD-LAMP to successfully detect *V*. *cholerae* presence down to 1 cell/reaction, which is 100-fold more sensitive than fluorescence-based measurements and similar to the detection sensitivity of next-generation signal transducer-based biosensors. We further show robust and rapid *V*. *cholerae* detection in complex sample matrices down to 10 cells/reaction using PD-LAMP. With these results, we envision that PD-LAMP will enable rapid and sensitive detection of other pathogens at low concentrations in their native environment.

## Results

### PD-LAMP Blinded Study

**Figure 2:**
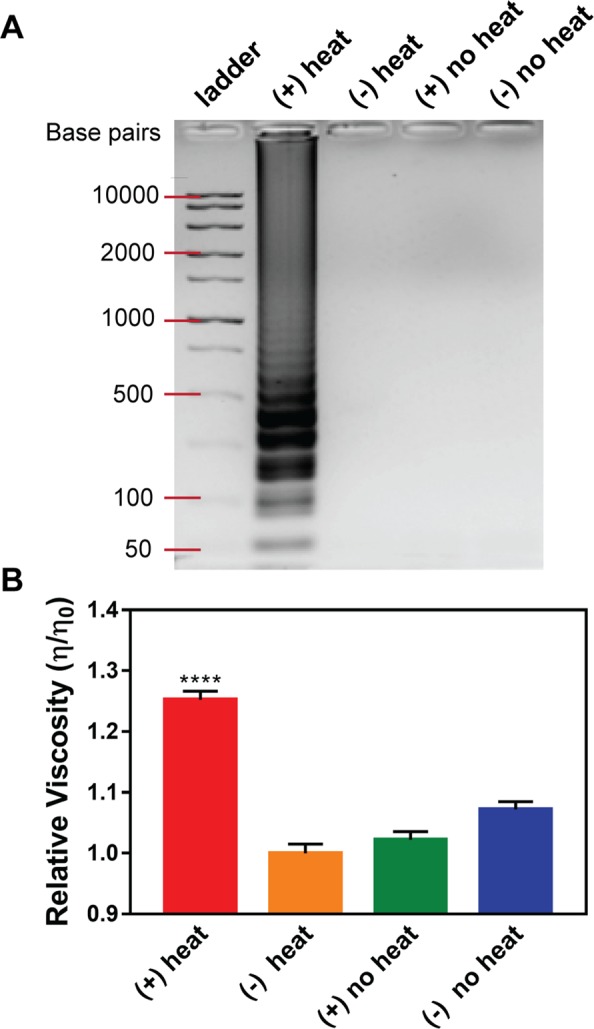
Relative viscosity blinded study. Here, genomic *V*. *cholerae* DNA that underwent the 65 °C heating of LAMP is represented as (+) heat, no genomic *V*. *cholerae* DNA that underwent the 65 °C heating of LAMP is (−) heat, genomic *V*. *cholerae* DNA that did not undergo heating is (+) no heat, and no genomic *V*. *cholerae* DNA that did not undergo heating is (−) no heat. (**A**) A 2% agarose gel of a representative blinded test to show DNA amplification in the positive sample. (**B**) Relative PD results show the experimental sample (+) heat is statistically significantly more viscous than control samples (****p-value < 0.0001). Nine PD measurements were made for each sample to allow for statistical comparison.

To validate that PD measurements of relative solution viscosity could be used to detect successful LAMP amplification, a series of blinded studies were performed. The sample containing the amplified genomic *V*. *cholerae* DNA was correctly identified with statistical significance (p-value < 0.0001) in every circumstance (n = 3). Data from one representative blinded study is presented in Fig. 2. The sample containing amplified genomic *V*. *cholerae* DNA had the greatest relative viscosity ($\eta /{\eta }_{0}$, calculated from equation (3)) as compared to the control samples, meaning that the presence of polymerized LAMP amplicons in the solution increased the fluid viscosity (Fig. 2B). Successfully identifying *V*. *cholerae* LAMP amplicons in blinded studies demonstrated that viscosity measurements are a feasible approach in determining pathogen presence.

### PD versus Fluorescence Measurements

**Figure 3:**
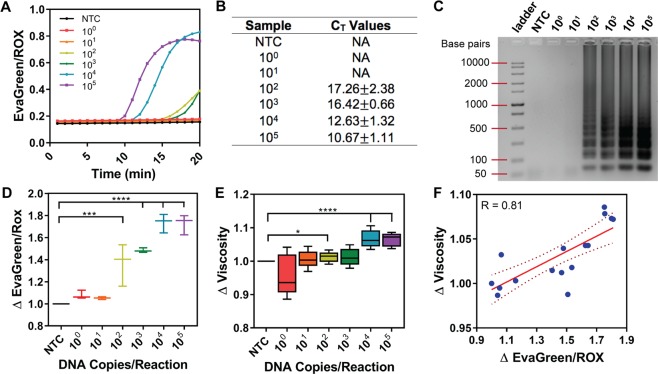
Detection of *V*. *cholerae* amplification using purified DNA. NTC represents no added *V*. *cholerae* DNA. (**A**) Real-time fluorescence was monitored over a 20-minute LAMP reaction for initial DNA concentrations between 10^0^–10^5^ DNA copies/reaction and (**B**) the corresponding C_T_ values were recorded for each reaction and are not available (NA) for samples that did not amplify. (**C**) A 2% agarose gel confirms amplification and presents the DNA banding pattern of LAMP amplicons at the different dilutions. (**D**) Box plots of the average change in fluorescence (${\rm{\Delta }}EvaGreen/Rox$) shows a trend of a greater change in fluorescence signal at higher initial *V*. *cholerae* DNA concentrations with statistical differences for samples 10^2^ (***p-value < 0.001), 10^3^, 10^4^, and 10^5^ (****p-value < 0.0001) DNA copies/reaction when compared to NTC. (**E**) Particle diffusometry measurements of the viscosity change of LAMP reactions show statistically significant measurements for 10^2^ (*p-value < 0.05), 10^4^, and 10^5^ (****p-value < 0.0001) DNA copies/reaction when compared to NTC. (**D**,**E**) Statistical analysis was a one-way ANOVA with Dunnett’s post-hoc relative to NTC (n = 3). (**F**) A positive correlation between change in fluorescence and PD yields a Pearson’s correlation coefficient of 0.81.

LAMP was performed with genomic *V*. *cholerae* DNA at concentrations ranging from 10^0^–10^5^ DNA copies/reaction (n = 3, data of repeats in Fig. S1). As expected, real-time visualization of the change in fluorescence showed that LAMP samples with higher initial concentrations of genomic *V*. *cholerae* DNA amplify more rapidly (Fig. 3A). Samples with lower concentrations showed slower, if any, amplification and resulted in a lower fluorescence signal at the end of the 20-minute period (corresponding C_T_ values are presented in Fig. 3B). Gel electrophoresis was used to confirm amplification of the DNA (Fig. 3C). The change in EvaGreen/Rox (${\rm{\Delta }}EvaGreen/Rox$), from the signal measured at 0 minutes and 20 minutes, was calculated by equation (4) (n = 3, Fig. 3D). The data indicated that a higher initial concentration of DNA corresponds with a greater ${\rm{\Delta }}EvaGreen/Rox$ signal at 20 minutes. We performed a one-way ANOVA with Dunnett’s post-hoc against the NTC sample and saw statistically significant differences for samples 10^2^, 10^3^, 10^4^, and 10^5^ DNA copies/reaction (p-value < 0.001 for 10^2^ and p-value < 0.0001 for 10^3^, 10^4^, and 10^5^ DNA copies/reaction).

PD was used to measure the change in the viscosity of *V*. *cholerae* DNA samples after the 20-minute LAMP reaction (data for individual repeats is presented in Fig. S1). Similar to the change in fluorescence, we found that as the initial concentration of *V*. *cholerae* DNA increased, there was a greater change in viscosity (${\rm{\Delta }}Viscosity$, Fig. 3E, calculated with equation (4)). Like the fluorescence measurements, statistically significant differences were seen between the ${\rm{\Delta }}Viscosity$ of NTC compared to 10^2^, 10^4^, and 10^5^ DNA copies/reaction (p-value < 0.05 and p-value < 0.0001, respectively) (Fig. 3E).

Correlation between the ${\rm{\Delta }}Viscosity$ (PD) and ${\rm{\Delta }}EvaGreen/Rox$ measurements confirmed agreement between the two methods. The correlation plot demonstrated in Fig. 3F with a calculated Pearson correlation coefficient (R) of 0.81 indicating that the two methods are strongly positively correlated with one another^28^. A slight discrepancy between measurements is expected since the polymerized DNA chains produced in each LAMP reaction vary in length^29^. Chain length has a major effect on solution viscosity^30^, and in turn the PD measurement^25^. However, the strong positive correlation between the two measurements demonstrated the feasibility of PD as a measurement technique for detection of *V*. *cholerae*.

### Measuring the Combined Effect of Changes in Particle Size and Viscosity with PD-LAMP

**Figure 4:**
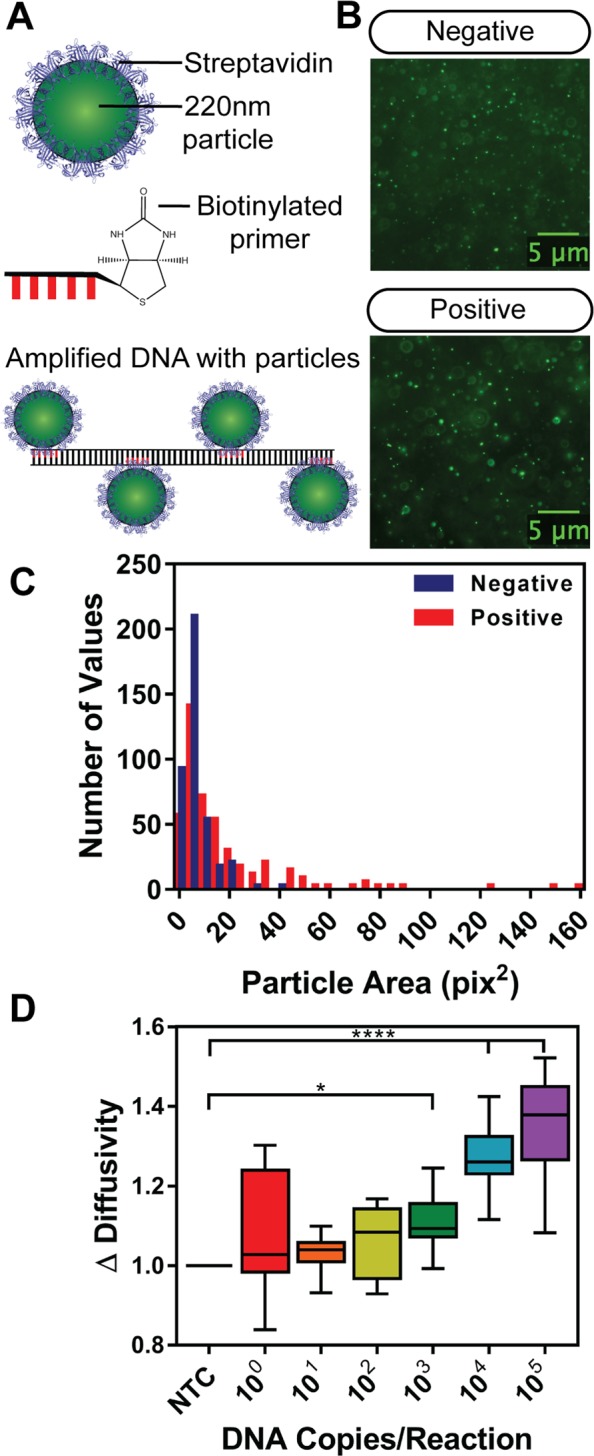
Biotinylated LAMP products measured using streptavidin conjugated particles. (**A**) Schematic of fluorescent streptavidin-coated polystyrene particles combined with DNA tagged with a biotinylated LAMP primer. (**B**) Representative image of fluorescent particles in a negative LAMP sample (top, no *V*. *cholerae* DNA). (**C**) Images in (**B**) are processed to quantify particle area (in pix^2^) for negative and positive LAMP reactions. (**D**) Measurement of diffusivity after amplification of various initial concentrations of *V*. *cholerae* DNA is statistically significant for 10^3^ (*p-value < 0.05), 10^4^, and 10^5^ (****p-value < 0.0001) DNA copies/reaction compared to NTC. Statistical analysis was a one-way ANOVA with Dunnett’s post-hoc relative to NTC (n = 4).

Despite successful detection of as few as 10^4^ DNA copies/reaction, we sought to improve the sensitivity of the of the PD measurements by combining detection of the change in viscosity with change in the hydrodynamic radius of the particles. Particle diffusivity (equation (2)) is a function of both viscosity of the solution ($\eta$), and the size of the measured particles ($a$). Similar to Tian *et al*.^31^, we used streptavidin conjugated polystyrene particles and biotinylated LAMP primer (LF) to bind the polymerized biotin-tagged DNA to the streptavidin polystyrene particles, creating multi-particle aggregates (Fig. 4A). First, we sought to experimentally validate that biotin-streptavidin induced aggregation occurs as a response only to *V*. *cholerae* DNA amplification in our system (Fig. 4B,C). In the negative sample (NTC), the streptavidin conjugated particles were uniformly distributed in the image (Fig. 4B, top). However, when particles were added to a positive sample after amplification, a cluster of particles was seen by the increase in fluorescence in regions of the image (Fig. 4B, bottom). Quantitative analysis showed that there was a narrower distribution of particle size around 10 pix^2^ in the negative samples and greater variability in the positive samples (Fig. 4C).

To quantify the relative change in particle motion upon changes in both viscosity and particle size, we measured the change in diffusivity, ${\rm{\Delta }}Diffusivity$, instead of the change in viscosity, ${\rm{\Delta }}Viscosity$ (equation (4)). We measured the ${\rm{\Delta }}Diffusivity$ in samples containing 10^0^–10^5^ DNA copies/reaction (n = 4, data of repeats in Fig. S2). The data showed a similar trend as observed in the uncoated beads; a higher initial DNA concentration leads to a higher ${\rm{\Delta }}Diffusivity$ measurement (Fig. 4D). Further, there was an increase in the baseline relative diffusivity when streptavidin conjugated particles were used compared to the uncoated 200 nm polystyrene beads (comparing the y-axis in Fig. 3E to Fig. 4D in which ${\rm{\Delta }}Diffusivity$ is directly proportional to ${\rm{\Delta }}Viscosity$). This baseline increase occurred as both the particle size and solution viscosity were considered (equation (2)). There was a statistically significant difference for the 10^3^, 10^4^ and 10^5^ DNA copies/reaction samples (p-value < 0.05 and p-value < 0.0001, one-way ANOVA with a post-hoc Dunnett’s test against NTC).

**Figure 5:**
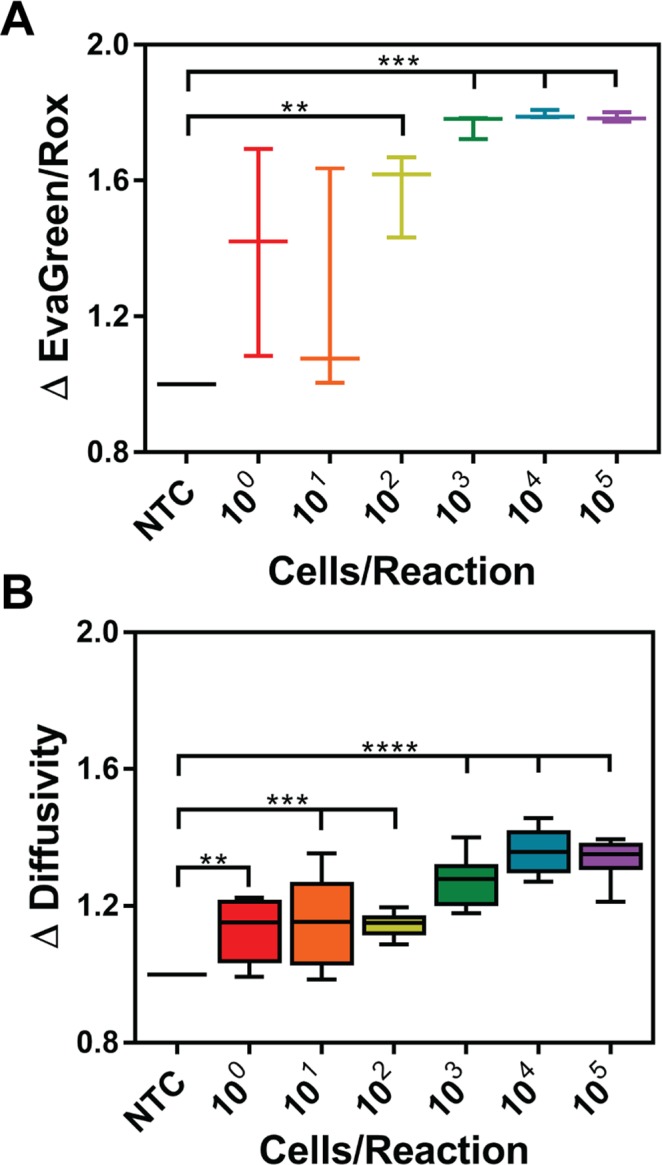
Measuring LAMP amplification from *V*. *cholerae* whole cells. Cells were spiked into LAMP reactions at concentrations ranging from 10^0^–10^5^ cells/reaction. The change in (**A**) EvaGreen/Rox is significant at 10^2^ (**p < 0.01), 10^3^, 10^4^, and 10^5^ (***p < 0.001) cells/reaction. (**B**) The change in diffusivity measurements show an increasing trend as a function of starting cell concentration with significance at 10^0^ (**p < 0.01), 10^1^, 10^2^ (***p < 0.001), 10^3^, 10^4^, and 10^5^ (****p < 0.0001) cells/reaction. Statistical analysis was a one-way ANOVA with Dunnett’s post-hoc relative to NTC (n = 3).

We wanted to determine if changes in diffusivity could be measured after amplification of DNA from whole cells. The cell lysate contained extra proteins that may potentially alter solution viscosity or cause changes in particle stability. It is important to note that *V*. *cholerae* cells, like other gram-negative bacteria, lysed due to thermal effects alone at 65 °C^32^, such that an additional cell lysis step beyond the LAMP assay was not necessary (validated in Fig. S3). LAMP assays with biotinylated primers and varying concentrations of whole *V*. *cholerae* cells (10^0^–10^5^ cells/reaction) were performed and the ${\rm{\Delta }}Diffusivity$ of streptavidin particles was measured with PD. As the initial concentration of *V*. *cholerae* cells increased, the ${\rm{\Delta }}EvaGreen/Rox$ and ${\rm{\Delta }}Diffusivity$ of the solutions also increased (Fig. 5). Real-time fluorescence curves and ${\rm{\Delta }}Diffusivity$ for each repeat are presented in Fig. S4. There was a statistically significant difference in the ${\rm{\Delta }}Diffusivity$ down to 10^0^ cells/reaction when compared to NTC (p-value < 0.01). In contrast, the ${\rm{\Delta }}EvaGreen/Rox$ measurements showed statistically significant differences down to 10^2^ cells/reaction when compared to NTC (p-value < 0.01). Interestingly, the PD-LAMP assay detected *V*. *cholerae* an order of magnitude lower using whole cells versus purified DNA (compare Figs 5B with 4D). These results are in agreement with Linnes *et al*. showing a 10-fold lower limit of detection when using isothermal amplification on whole cell *Chlamydia trachomatis* samples as compared to purified corresponding DNA^33^. Relative to fluorescence measurements, the PD measurements were 100-fold more sensitive (comparing Fig. 5A,B). This is promising in the implementation of PD as a pathogen detection technique considering that environmental samples collected for testing would contain very low concentrations of *V*. *cholerae* cells.

### Environmental Water Sources

*V*. *cholerae* is a pathogen found in environmental water sources^34,35^, thus it is essential to perform PD-LAMP on cells in complex matrices other than molecular biology water. PD-LAMP was performed in 1X PBS, tap water, rain runoff, and pond water (and molecular biology water as a control) with 10^5^*V*. *cholerae* cells spiked into each 25 µL reaction (positive samples) or with no cells (negative control) for each water source. These water sources presented three potential challenges for accurate PD detection of DNA amplification: (1) inhibition of the LAMP assay, (2) adverse effects to the particles during the measurement including degradation or aggregation, and (3) unaccounted changes that may occur due to excess particulates that may non-specifically bind to the particles by increasing apparent size or create viscosity changes.

**Figure 6:**
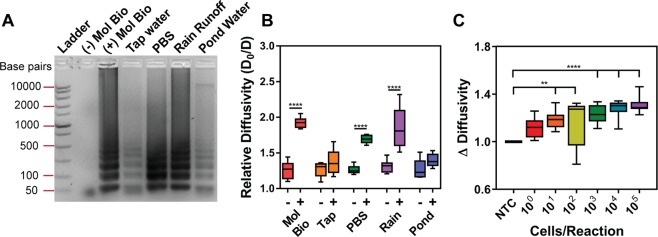
LAMP amplification from whole *V*. *cholerae* cells in environmental water sources. Different water sources were used in the LAMP reactions for (**A**) and (**B**). Molecular biology water (Mol Bio) was used as a control ((−) no *V*. *cholerae* cells, (+) *V*. *cholerae* cells) for each water source in (**A**) and (**B**). (A) Gel electrophoresis shows less DNA amplification in pond and tap water groups compared to other water sources. (**B**) PD measurement of relative diffusivity shows a statistically significant difference between negative and positive samples for molecular biology water, PBS, and rain runoff groups (****p-values < 0.0001). (**C**) *V*. *cholerae* cells were spiked into pond water at concentrations ranging from 10^0^–10^5^ cells/reaction. The change in diffusivity measurements show an increasing trend as a function of starting cell concentration with significance at 10^1^, 10^2^ (**p < 0.01), 10^3^, 10^4^, and 10^5^ (****p < 0.0001) cells/reaction. Statistical analysis was a one-way ANOVA with Dunnett’s post-hoc relative to NTC (n = 3).

Gel electrophoresis (Fig. 6A) and quantitative fluorescence measurements (Fig. S5) showed little DNA amplification in tap water. We measured the relative diffusivity using PD to investigate this difference (${D}_{0}/D$, equation (3), repeat data presented in Fig. S5). There was no statistically significant difference in relative diffusivity of positive samples in tap water compared to the negative controls (Fig. 6B, p-value > 0.05, student t-test). This was to be expected because tap water contains chlorine, which likely inhibited the activity of the Bst 2.0 enzyme required for the LAMP assay^36^.

Since *V*. *cholerae* can be harbored in sea water, we performed LAMP in 1X PBS to determine whether salt affects the LAMP assay or PD measurements. LAMP assays performed in 1X PBS did not show any inhibition when analyzing both fluorescence measurements and gel electrophoresis (Figs 6A and S5). Similarly, there was a statistically significant difference in PD measurements (p-value < 0.0001, student t-test) between the negative and positive PBS samples. This confirmed that salt content did not inhibit the LAMP assay or PD measurements (Fig. 6B).

Using PD, we analyzed LAMP assays performed in rain runoff and pond water, both which contained sediment and therefore added to sample matrix complexity. PD measurements showed a statistically significant difference in the relative diffusivity of rain runoff samples with *V*. *cholerae* cells compared to its negative control (Fig. 6B, p-value < 0.0001, student t-test). This is extremely promising given that the water source was collected outside of the laboratory space and potentially contained particulates that inhibit the LAMP reaction. In contrast, LAMP assays in pond water demonstrated a decrease in amplification signal (Fig. S5). This was supported with the faded banding pattern in the agarose gel (Fig. 6A). Due to the minimal amplification, PD reflected little-to-no change in the diffusivity between the negative and positive *V*. *cholerae* samples in pond water (Fig. 6B, p-value > 0.05, student t-test).

Further characterization of PD-LAMP in pond water was performed, as this source is the best surrogate of the native environment for *V*. *cholerae*^37,38^. LAMP assays with biotinylated primers and varying concentrations of whole *V*. *cholerae* cells (10^0^–10^5^ cells/reaction) were performed in pond water (where 50% of the total LAMP reaction volume was pond water). The ${\rm{\Delta }}Diffusivity$ of streptavidin particles in the presence of the LAMP reaction products was measured with PD. Because the debris and ions in pond water slightly inhibit nucleic acid amplification reactions^39^, the LAMP assay was run for 35 minutes. As the initial concentration of *V*. *cholerae* cells increased, the ${\rm{\Delta }}EvaGreen/Rox$ and ${\rm{\Delta }}Diffusivity$ of the solutions also increased (Fig. 6C). There was a statistically significant difference in the ${\rm{\Delta }}Diffusivity$ down to 10 cells/reaction when compared to NTC (p-value < 0.01). Real-time fluorescence curves and ${\rm{\Delta }}Diffusivity$ for each repeat are presented in Fig. S6. In contrast, the ${\rm{\Delta }}EvaGreen/Rox$ measurements showed statistically significant differences down to 10^2^ cells/reaction when compared to NTC (p-value < 0.05) (Fig. S7). Relative to fluorescence measurements, the PD measurements were 10-fold more sensitive (comparing Figs 6C to S7).

## Discussion

In this work, we demonstrate that PD-LAMP can be used as a rapid, sensitive, and robust method for the detection of *V*. *cholerae* in environmental water samples. In blinded studies PD-LAMP could detect the presence of *V*. *cholerae* DNA with 100% accuracy (Fig. 2B). Additionally, there is a strong, positive correlation (R = 0.81) between ${\rm{\Delta }}EvaGreen/Rox$ (quantitative fluorescence) and ${\rm{\Delta }}Viscosity$ (PD measurements). Our studies indicate that PD-LAMP can detect as few as 1 *V*. *cholerae* cell/reaction in molecular-grade water (Fig. 5B) which is 100-fold more sensitive than gold standard fluorescence measurements^1,40^. Furthermore, we demonstrate that PD-LAMP is robust enough to detect down to 10 cells/reaction of *V*. *cholerae* DNA from cells lysed *in situ* during a 35-minute reaction in pond water without the need for additional sample preparation (Fig. 6C). This detection method is 10-fold more sensitive than current gold standard fluorescence detection of nucleic acid amplification. This is the first study directly comparing fluorescence detection and the novel PD-LAMP method. We have demonstrated that PD-LAMP offers at least a 10-fold increase in sensitivity over fluorescence detection. These results establish the utility of combining both changes in size and viscosity for improved signal-to-noise measurements with PD-LAMP for the rapid detection of *V*. *cholerae*. PD is an alternate method to fluorescence detection for nucleic acid amplification products that has a significant improvement in sensitivity and is robust enough to detect the amplified products in their native sample matrix.

PD-LAMP as a biosensor is pathogen agnostic; it is not limited to *V*. *cholerae* identification, but it can serve as a platform for the detection of a wide variety of pathogens. Due to the success in detecting *V*. *cholerae*, we envision PD-LAMP as an effective detection method to identify pathogenic DNA for other infectious diseases including *E*. *coli* and salmonella. PD-LAMP is particularly attractive due to the passive nature of the detection method (i.e. optically detecting Brownian motion) compared to current DNA detection techniques that require chemical reactions involving fluorophore intercalation, colorimetric, or turbidimetric readouts derived from magnesium products, or electrochemical techniques. Future development of PD-LAMP would involve designing the biosensor as a handheld device. This is plausible considering that PD-LAMP involves only a microscope, camera, and computer. Miniaturization and integration of these components would allow for the translation of a field deployable biosensor for pathogen detection.

## Methods

### Bacteria Culture

The *V*. *cholerae* strain N16961, a toxigenic O1 serogroup, was provided by Dr. Afsar Ali, from the Department of Environmental and Global Health at the University of Florida. All cultures were grown aerobically in Lysogeny Broth (LB) overnight at 37 °C using a miniature incubating shaker at 300 rpm (Thermo Fisher, Waltham, MA). Cultures were diluted in LB media to an OD_600_ of 1, (Ultrospec 10, Biochrom, Cambourne, UK) representing 6 × 10^8^ cells/mL of *V*. *cholerae* as determined by counting colony forming units of serially diluted samples.

### Loop-Mediated Isothermal Amplification (LAMP)

Purified genomic DNA from *V*. *cholerae* N16961 (American Type Culture Collection 39315D-5, Manassas, VA) was maintained in 2.2 ng/µL aliquots for use in preliminary testing and at specified concentrations in experiments thereafter. LAMP primers were devised to target the cholera toxin A (*ctxA*) gene within the toxigenic *V*. *cholerae* strain^23^. There is one *ctxA* gene copy per genome. The primer sequences are provided in Table S1 Supplemental. For all amplification experiments, 25 µL reactions consisted of 23 µL of LAMP master mix (components shown in Table S2 Supplemental). 2 µL of sample (purified *V*. *cholerae* DNA or whole cells) or negative control (molecular biology water (Invitrogen, Carlsbad, CA)) was added just prior to heating. LAMP was performed at 65 °C for 20, 25, or 35 minutes using an Applied Biosystems 7500 Real-Time PCR System (Foster City, CA), and then the samples were stored at 4 °C until analyzed with PD.

LAMP was performed with *V*. *cholerae* purified genomic DNA or whole cells. 10-fold serial dilutions of template (both DNA and cells) were prepared for experimentation (10^0^ to 10^5^ copies of DNA or cells per reaction) in molecular biology water. Real-time fluorescent data was collected for each experiment to visually track the amplification progress. LAMP amplicons were also characterized via gel electrophoresis using a 2% agarose gel at 100 V for 60 minutes, stained with ethidium bromide, and imaged using an ultraviolet light gel imaging system (c400, Azure Biosystems, Dublin, CA). All gel images were collected using the Azure cSeries software and settings of UV302 with an exposure time of 15 seconds. The gel electrophoresis images, exported as JPG files, were not cropped nor edited.

### Particle Preparation

For viscosity measurements, red fluorescent 200 nm polystyrene particles (Fluoro-max red dyed aqueous spheres, Thermo Scientific, Erie, NY, USA) were combined with the LAMP products. Polystyrene particles were chosen due because they are similar in density to water, making them relatively neutrally buoyant, allowing the effects of gravity to remain negligible for particle diffusometry measurements. Further, the 200 nm particle size was chosen to achieve more sensitive measurements as smaller particles exhibit greater changes in diffusivity These particles were washed three times in MilliQ water by centrifugation at 13,000 x g for 15 minutes. Following, the particles were added to the LAMP products at a final concentration of 6 × 10^9^ particles/mL and stored at 4 °C until imaging.

For combined size and viscosity measurements (i.e. diffusivity), streptavidin coated 220 nm green polystyrene fluorescent particles were used (Bangs Labs, Fishers, IN, USA) to maintain a particle diameter as close as possible to the 200 nm unmodified polystyrene particles used for viscosity measurements. Due to supplier constraints, 220 nm was the nearest particle diameter with streptavidin-modified surface chemistry. Particles were washed three times in MilliQ water by centrifugation at 13,000 x g for 15 minutes. Washed particles were added to the LAMP products at a final concentration of 1.49 × 10^9^ particles/mL (note that the final concentrations of the 200 and 220 nm particle samples differ due to their size to eliminate hindered diffusion). Particles and LAMP amplicons were incubated at 4 °C by gentle rotation for two hours to allow binding of the biotinylated LF primer to the streptavidin particles and then imaged.

### Particle Diffusometry Theory

PD involves recording a series of images of fluorescent particles undergoing Brownian motion in a quiescent volume and calculating the particle diffusion coefficient using correlation analysis^24^. Each individual image is partitioned into smaller interrogation areas where the size of each interrogation area is defined such that 8–10 particles, on average, are present^41^. Within these interrogation areas, autocorrelations and cross-correlations of the images are computed for the entire image stack. Cross-correlation involves correlating two sequential images, for example taken at time $t$ and at time $t+{\rm{\Delta }}t$ (where ${\rm{\Delta }}t$ is a function of the frame rate). Greater particle displacement, during the elapsed time ${\rm{\Delta }}t$, creates broader cross-correlation peaks^42^. The cross-correlation peak width, ${s}_{c}$ (pixels) at a height of $1/e$, is used to calculate the diffusion coefficient^43^. Further, autocorrelation is performed by correlating an image captured at time $t$ with itself. The autocorrelation peak width, ${s}_{a}$ at a height of $1/e$, is taller and narrower when compared to the cross-correlation peak^42^. With autocorrelation and cross-correlation, the diffusion coefficient can be calculated by the equation derived by Olsen and Adrian^44^:

1 $$D=\frac{{s}_{c}^{2}-{s}_{a}^{2}}{16{M}^{2}{\rm{\Delta }}t}$$

where *M* is the magnification of the microscope objective. Because the peak width is in units of pixels, using equation (1), we see that the squared difference in the peak widths, ${s}_{c}^{2}-{s}_{a}^{2}$, corresponds to the change in the cross-sectional area of the correlation peak at $1/e$. By experimentally determining the diffusion coefficient, *D*, from the series of particle images, the Stokes-Einstein relationship can be algebraically rearranged (equation (2)) to calculate the viscosity, η, of a solution^45,46^.

2 $$\eta =\frac{kT}{6\pi Da}$$

Here, $k$ is the Boltzmann constant, $T$ is the absolute temperature, and $a$ is the hydrodynamic radius of fluorescent spheres that are imaged. It is important to note that smaller diameter particles will provide a greater signal-to-noise ratio in solutions where there are only modest changes in viscosity.

We are specifically interested in characterizing how the presence of LAMP amplicons affects diffusivity of particles for pathogen detection. The change in diffusivity in this context are due to changes in solution viscosity and/or particle size. Therefore, we compute the relative solution viscosity or the combined relative size and solution viscosity, forgoing the magnitude in either case. Algebraic manipulation of equation (2), where ${\eta }_{0}$ is the viscosity of the buffer solution without the LAMP amplicons (but still including LAMP primers and fluorescent particles), produces the relative viscosity ($\eta /{\eta }_{0}$).

3 $$\frac{\eta }{{\eta }_{0}}=\frac{\eta a}{{\eta }_{0}{a}_{0}}=\frac{{D}_{0}}{D}$$

Further, the equation (2) can be manipulated to include relative size. In equation (3), ${a}_{0}$ is the size of nanoparticles where no DNA is amplified and $a$ is the size of the particles after amplification. This approach is optimal in binary situations, where an investigator is interested in the presence or absence of pathogens in a solution.

Sample-to-sample variation often occurs in the quantitative measurements of LAMP assays due to the polymerization process^29^. Therefore, when measuring the change in the diffusion coefficient as a function of the concentration of LAMP amplicons, we calculate the change (${\rm{\Delta }}$) in the signal. This approach is used when comparing real-time LAMP fluorescence measurements with PD viscosity measurements. In equation (4) the signal change (${\rm{\Delta }}Signal$, either fluorescence or viscosity) is a function of the signal after amplification ($final\,measurement$) and before amplification ($initial\,measurement$).

4 $${\rm{\Delta }}Signal=\frac{final\,measurement-initial\,measurement}{final\,measurement}+1$$

### Experimental Particle Diffusometry Measurements

A fluid well for the LAMP-particle solution was made by punching a 6 mm diameter hole (120 μm thickness) through double-sided adhesive (Therm-O-Web, Wheeling, IL, USA) which was then adhered to a cover glass slide (Thickness No. 1, Thermo Scientific, Erie, NY, USA). The 3 μL LAMP-particle solution was added to the fluid well and sealed with a second cover glass slide to limit convective evaporation during imaging.

The LAMP-particle solutions were imaged at room temperature using an inverted fluorescence microscope (Nikon TE-2000U, Nikon, Japan) equipped with an X-cite lamp and 40X magnification objective using PCO Camware software (PCO, Kelheim, Germany). Images were recorded using a PCO 1600 CCD camera (PCO, Kelheim, Germany) using a 802 × 802 pixel^2^ imaging window with 2 × 2 binning at 13.3 fps. Individual pixels were 7.4 × 7.4 μm^2^. For imaging of the 200 nm red polystyrene spheres, a Q-Dot 585 filter cube was used (Chroma, Bellows Falls, VT) and for the 220 nm green polystyrene spheres, a B3-A filter cube was used (Nikon, Japan).

Particles were imaged at the mid-plane of the chip to ensure the effects of hindered diffusion caused by the proximity of particles to any wall was avoided. We analyzed particles which were located in the depth of correlation, 4.2 μm, by using an expression derived by Meinhart *et al*.^47,48^. Therefore, particles located within the depth of correlation form the correlation function and the remainder of particles contribute to the background signal.

Particle images were processed and auto- and cross-correlation analysis was performed using an in-house MATLAB code. 64 × 64 pixel^2^ interrogation areas contain, on average, 8–10 particles per 100 image frame stacks (~8 seconds of data). This allowed for a high signal-to-noise ratio while maintaining a statistically relevant number of data points. Nine measurements, of which 100 images constituted one measurement, were performed for every sample. A two-dimensional Gaussian curve fit was used to calculate the auto- and cross-correlation peak widths for both the XZ- and YZ-planes. The width of the correlation peak is defined by $1/e$ and the width of the XZ- and YZ-Gaussian curves are averaged as one peak width value^43^.

### Blinded Study

Control samples contained (1) no *V*. *cholerae* DNA that underwent the 65 °C heating for 20 minutes ((−) heat), (2) genomic *V*. *cholerae* DNA that did not undergo heating ((+) no heat), and (3) no *V*. *cholerae* DNA that did not undergo heating ((−) no heat). The experimental sample contained genomic *V*. *cholerae* DNA and was amplified at 65 °C for 20 minutes ((+) heat). The four samples were unknown to the researcher performing the PD testing in order to obtain unbiased measurements. The four samples contained 200 nm red fluorescent unmodified polystyrene spheres that were added to the samples after amplification and were imaged under fluorescence microscopy (data in Table S3 Supplemental). Data was represented in terms of relative viscosity ($\eta /{\eta }_{0}$, equation (3)).

### Water Testing

All LAMP reactions in various water sources were prepared using the standard master mix reagents with the biotinylated LF primer and were performed for 25 minutes. The different water sources were 50% by volume of the LAMP reaction. Water sources included laboratory tap water, autoclaved 1X phosphate buffered-saline (PBS) (Sigma-Aldrich, St. Louis, MO) pH 7.4, rain runoff, pond water, and molecular biology water. Rain runoff and pond water collected from the bank of a small stagnant pond were stored at 4 °C until LAMP was performed. In the experiments that investigated the limit of detection of whole *V*. *cholerae* cells in pond water, the LAMP assay was run for a total of 35 minutes due to the slight delay caused by the inhibitors in pond water^39^.

### Statistical Analysis

The blinded test groups were statistically analyzed with a one-way ANOVA with multiple comparisons using a 95% confidence interval, comparing data represented in terms of relative viscosity ($\eta /{\eta }_{0}$, equation (3)). In measuring the 10-fold dilutions and determining the LOD, data was represented in terms of ${\rm{\Delta }}Viscosity$ or ${\rm{\Delta }}Diffusivity$ (equation (4)). When comparing a series of 10-fold dilutions, a one-way ANOVA post-hoc Dunnett’s test was performed with multiple comparisons against a negative control with no template (no template control, NTC) with a 95% confidence interval. To compare ${\rm{\Delta }}Fluorescence$ and ${\rm{\Delta }}Viscosity$, the Pearson’s correlation coefficient was calculated. A Student’s paired t-test with a 95% confidence interval was used when comparing the negative control and positive samples in different water types, with data again represented in terms of relative viscosity ($\eta /{\eta }_{0}$, equation (3)). Box-and-whisker plots were made for the PD and fluorescent data for the 10-fold dilutions where the upper and lower bounds represent the 75^th^ and 25^th^ percentile about the median, respectively, and the minimum and maximum values represented by the upper and lower whiskers.

## Supplementary information

Electronic Supplementary Information
