# Supplementary material for: Particle Diffusometry: An Optical Detection Method for Vibrio cholerae Presence in Environmental Water Samples
Source: Sci Rep. 2019 Feb 11;9:1739. doi: 10.1038/s41598-018-38056-7 (PMC6370876; doi:10.1038/s41598-018-38056-7)
Supplement: Supplementary file 1 — Electronic Supplementary Information [file 41598_2018_38056_MOESM1_ESM.pdf]

Electronic Supplementary Information

Particle Diffusometry: An Optical Detection Method for *Vibrio cholerae* Presence in Environmental Water Samples

Katherine N. Clayton,[a†] Taylor J. Moehling,[b†] Dong Hoon Lee,[a] Steven T. Wereley,[a] Jacqueline C. Linnes,[b*] Tamara L. Kinzer-Ursem[b*]

[a] *School of Mechanical Engineering, Purdue University, West Lafayette, IN 47906*

[b] *Weldon School of Biomedical Engineering, Purdue University, West Lafayette, IN 47906*

E-mail: jlinnes@purdue.edu (J.C. Linnes), tursem@purdue.edu (T.L. Kinzer-Ursem)

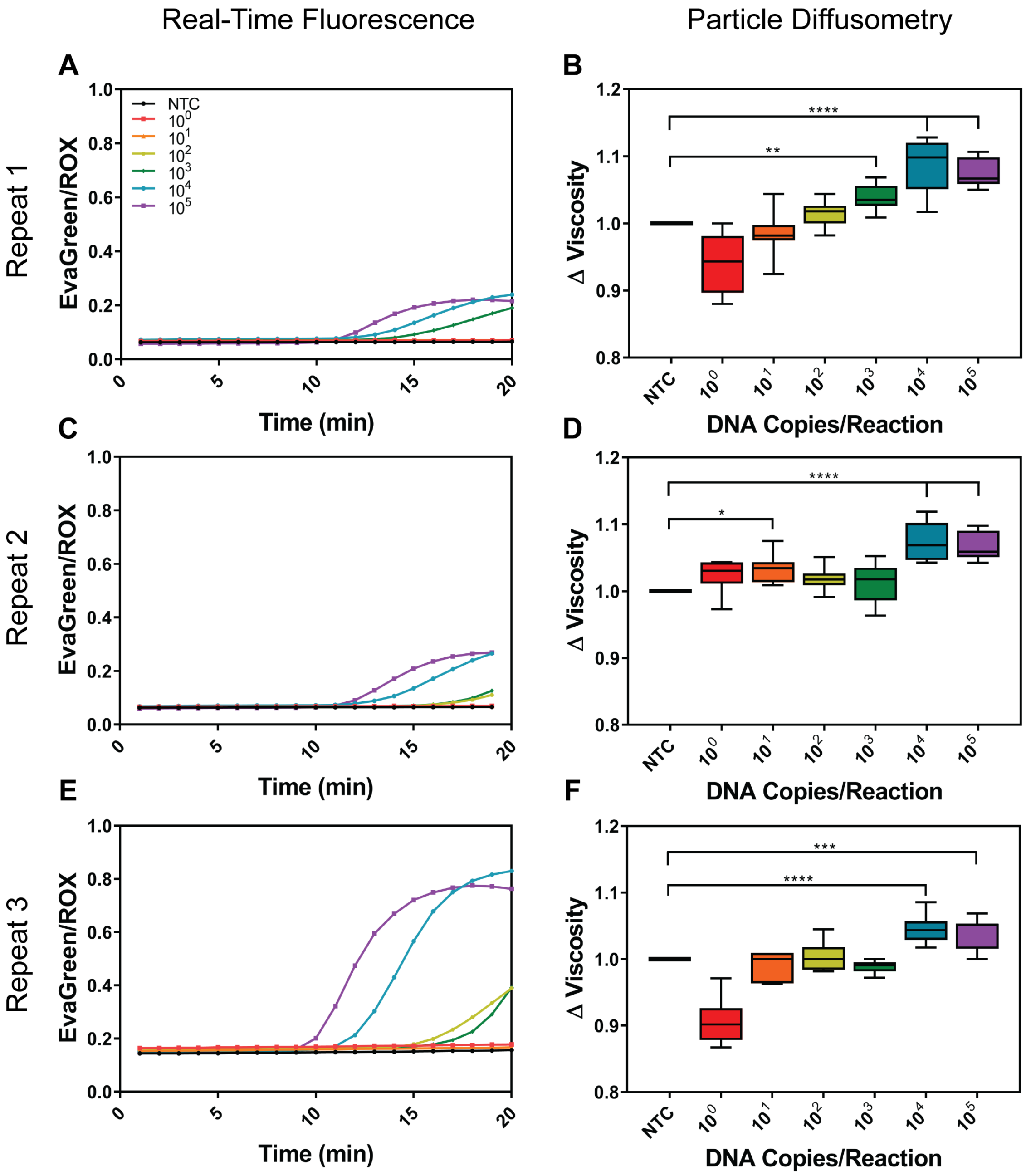

Fig. S1. Real-time fluorescence and PD measurements for DNA dilutions. (A) Real-time fluorescent curves show amplification for different concentrations of DNA for repeat 1. (B) PD measurements were performed on the same samples as in (A). PD measurements of viscosity change for the 10^3, 10^4, and 10^5 DNA copies/reaction samples are statistically significantly different relative to NTC (** p<0.01, **** p<0.0001, and **** p<0.0001, respectively). (C) Real-time fluorescent amplification curves for DNA concentrations for repeat 2. (D) PD measurements of the same samples as in (C). PD measurements of viscosity change for the 10^4 and 10^5 DNA copies/reaction samples are statistically significantly different relative to NTC (**** p<0.0001, and *** p<0.001, respectively). (E) Real-time fluorescent curves for different concentrations of DNA for repeat 3. (F) PD measurements of the same samples as in (E). PD measurements of viscosity change for the 10^2, 10^4, and 10^5 DNA copies/reaction samples are statistically significantly different relative to NTC (* p<0.05, **** p<0.0001, and **** p<0.0001, respectively). A one-way ANOVA post-hoc Dunnett's test was performed for all repeats with multiple comparisons against NTC with a 95% confidence interval.

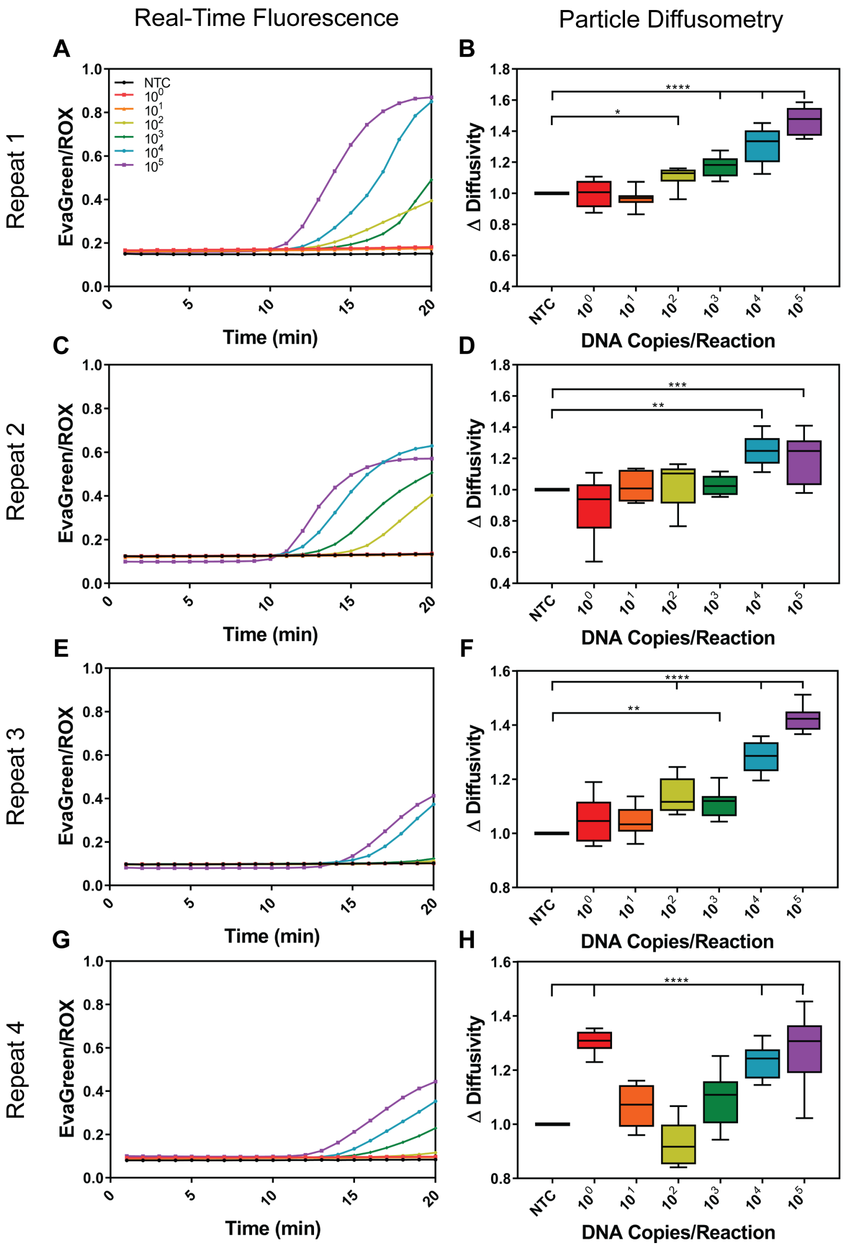

Fig. S2. Real-time fluorescence and PD measurements for biotinylated DNA dilutions. (A) Real-time fluorescent curves show amplification for different concentrations of DNA for repeat 1. (B) PD measurements were performed on the same samples as in (A). PD measurements of diffusivity change for the 10^2, 10^3, 10^4, and 10^5 DNA copies/reaction are statistically significantly different relative to NTC (* $p<0.05$, **** $p<0.0001$, **** $p<0.0001$, and **** $p<0.0001$, respectively). (C) Additional real-time fluorescent curves for repeat 2. (D) PD measurements were performed on the same samples as in (C). PD measurements of diffusivity change for the 10^4, and 10^5 DNA copies/reaction are statistically significantly different relative to NTC (** $p<0.01$, and *** $p<0.001$, respectively). (E) Repeat 3 real-time fluorescent curves. (F) PD measurements were performed on the same samples as in (E). PD measurements of diffusivity change for the 10^2, 10^3, 10^4, and 10^5 DNA copies/reaction are statistically significantly different relative to NTC (**** $p<0.0001$, ** $p<0.01$, **** $p<0.0001$, and **** $p<0.0001$, respectively). (G) Repeat 4 real-time fluorescent curves. (H) PD measurements were performed on same samples as in (G). PD measurements of diffusivity change for the 10^0, 10^4, and 10^5 DNA copies/reaction are statistically significantly different relative to NTC (**** $p<0.0001$, **** $p<0.0001$, and **** $p<0.0001$ respectively). A one-way ANOVA post-hoc Dunnett's test was performed for all repeats with multiple comparisons against NTC with a 95% confidence interval.

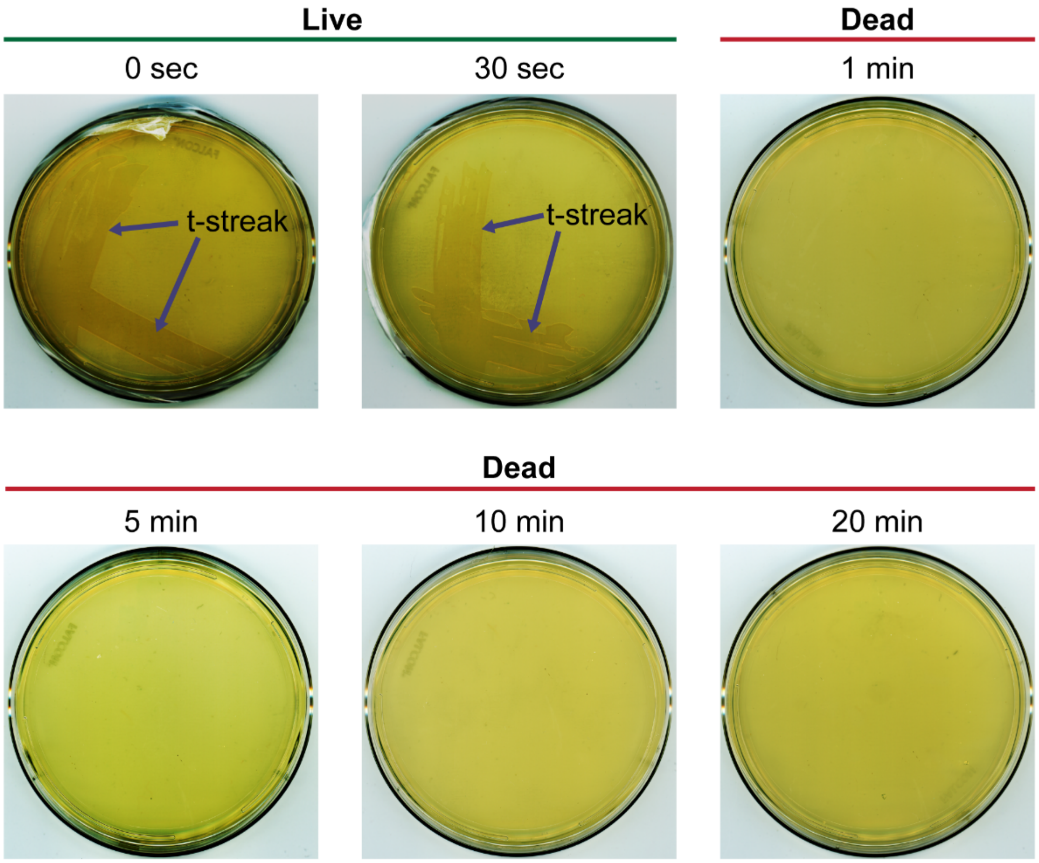

Fig. S3. *Vibrio cholerae* thermal cell lysis at 65°C. 5 mL LB culture of *V. cholerae* was grown overnight at 37°C and diluted to OD_{600} of 1. 25 μL aliquots of the overnight culture were subject to heating at 65°C for various lengths of time (0 sec, 30 sec, 1 min, 5 min, 10 min, and 20 min) and then immediately streaked onto a LB agar plate. After a 9-hour incubation period at 37°C, images were taken on an Epson Perfection V850 Pro Scanner (Suwa, Japan). Based on the time-series images above, *V. cholerae* cell lysis occurs between 30 second and 1 minute of heating. This is can be seen from the cluster of colonies seen in the 0 and 30 second plates (marked by "t-streak" in the image) and the absence of these colonies at heating times greater than 1 minute.

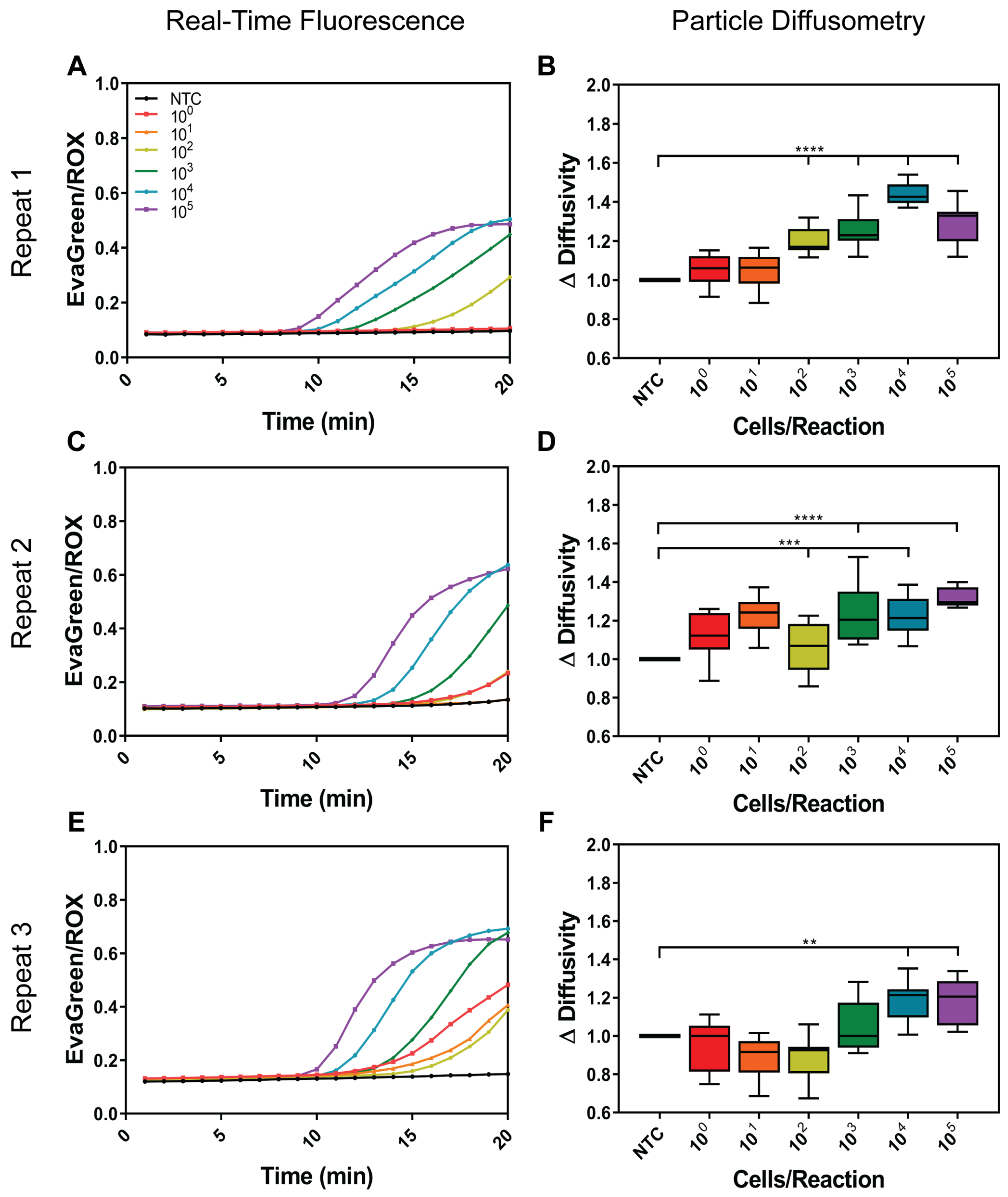

Fig. S4. Real-time fluorescence and PD measurements for biotinylated DNA from whole *V. cholerae* cell dilutions. (A) Real-time fluorescent curves show amplification for different concentrations of cells for repeat 1. (B) PD measurements were performed on the same samples as in (A). PD measurements of diffusivity change for the 10^2, 10^3, 10^4, and 10^5 cells/reaction are statistically significantly different relative to NTC (**** $p<0.0001$, for all). (C) Repeat 2 real-time fluorescent curves. (D) PD measurements were performed on the same samples as in (C). PD measurements of diffusivity change for the 10^2, 10^3, 10^4, and 10^5 cells/reaction are statistically significantly different relative to NTC (*** $p<0.001$, **** $p<0.0001$, *** $p<0.001$, and **** $p<0.0001$, respectively). (E) Repeat 3 real-time fluorescent curves. (F) PD measurements were performed on the same samples as in (E). PD measurements of diffusivity change for the 10^4 and 10^5 cells/reaction are statistically significantly different relative to NTC (** $p<0.01$ for both). A one-way ANOVA post-hoc Dunnett's test was performed for all repeats with multiple comparisons against NTC with a 95% confidence interval.

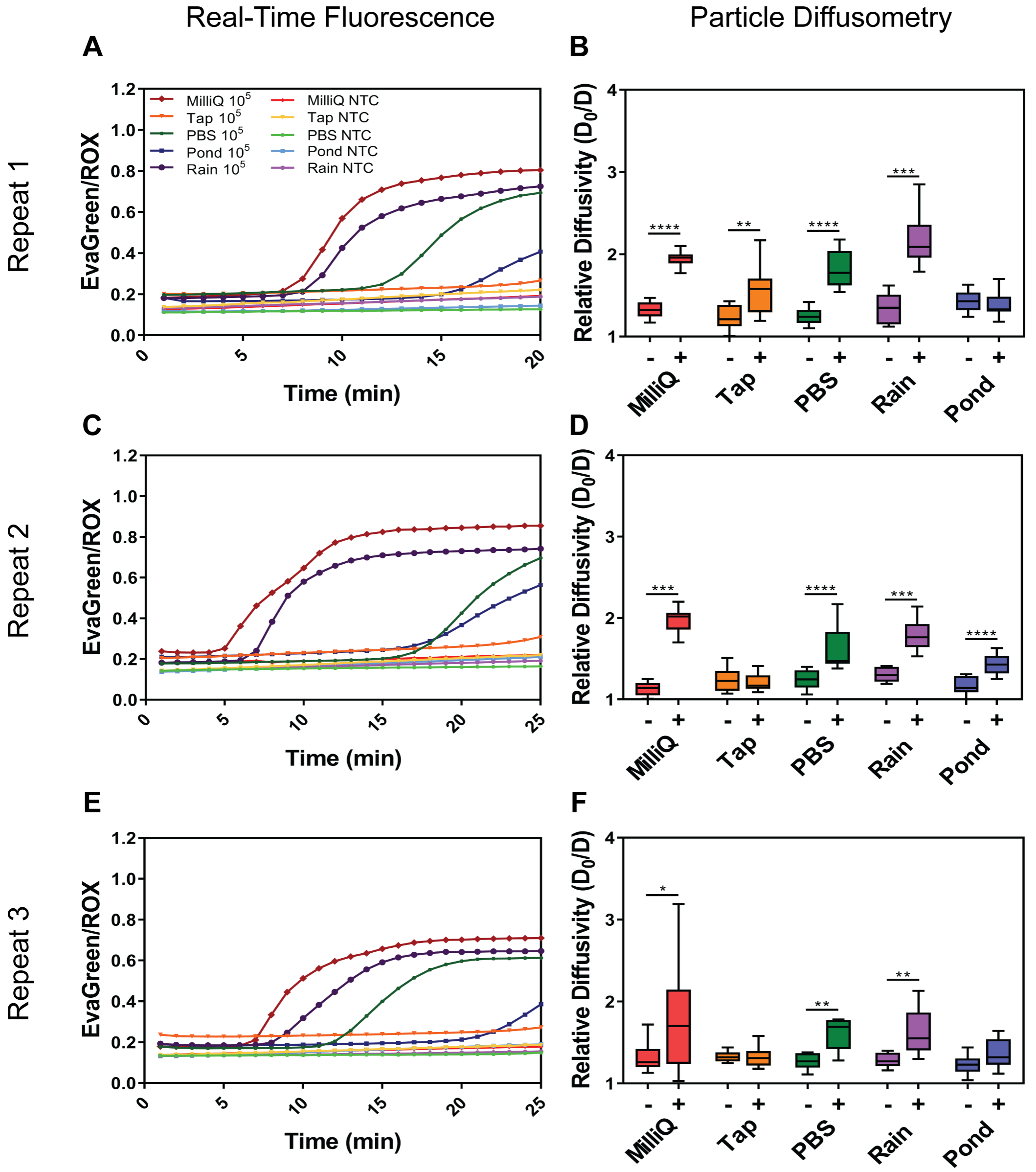

Fig. S5. Real-time fluorescence and PD measurements for biotinylated DNA from whole *V. cholerae* cells spiked in various water types. (A) Real-time fluorescent curves. (B) PD measurements were performed on the same samples as in (A). PD measurements of relative diffusivity for MilliQ, Tap, PBS, and Rain with cells (+) are statistically significantly different relative to their respective negative (-) (**** p<0.0001, ** p<0.01, **** p<0.0001, *** p<0.001, respectively). (C) Real-time fluorescent curves show amplification in various water types for repeat 2. (D) PD measurements were performed on the same samples as in (C). PD measurements of relative diffusivity for MilliQ, PBS, Rain, and Pond (+) are statistically significantly different relative to their respective negative (-) (*** p<0.001, **** p<0.0001, *** p<0.001, **** p<0.0001, respectively). (E) Real-time fluorescent curves show amplification in various water types for repeat 3. (F) PD measurements were performed on the same samples as in (E). PD measurements of relative diffusivity for MilliQ, PBS, and Rain (+) are statistically significantly different relative to their respective negative (-) (* p<0.05, ** p<0.01, ** p<0.01, respectively). A Student's paired t-test with a 95% confidence interval was used when comparing the negative control and positive samples in different water types.

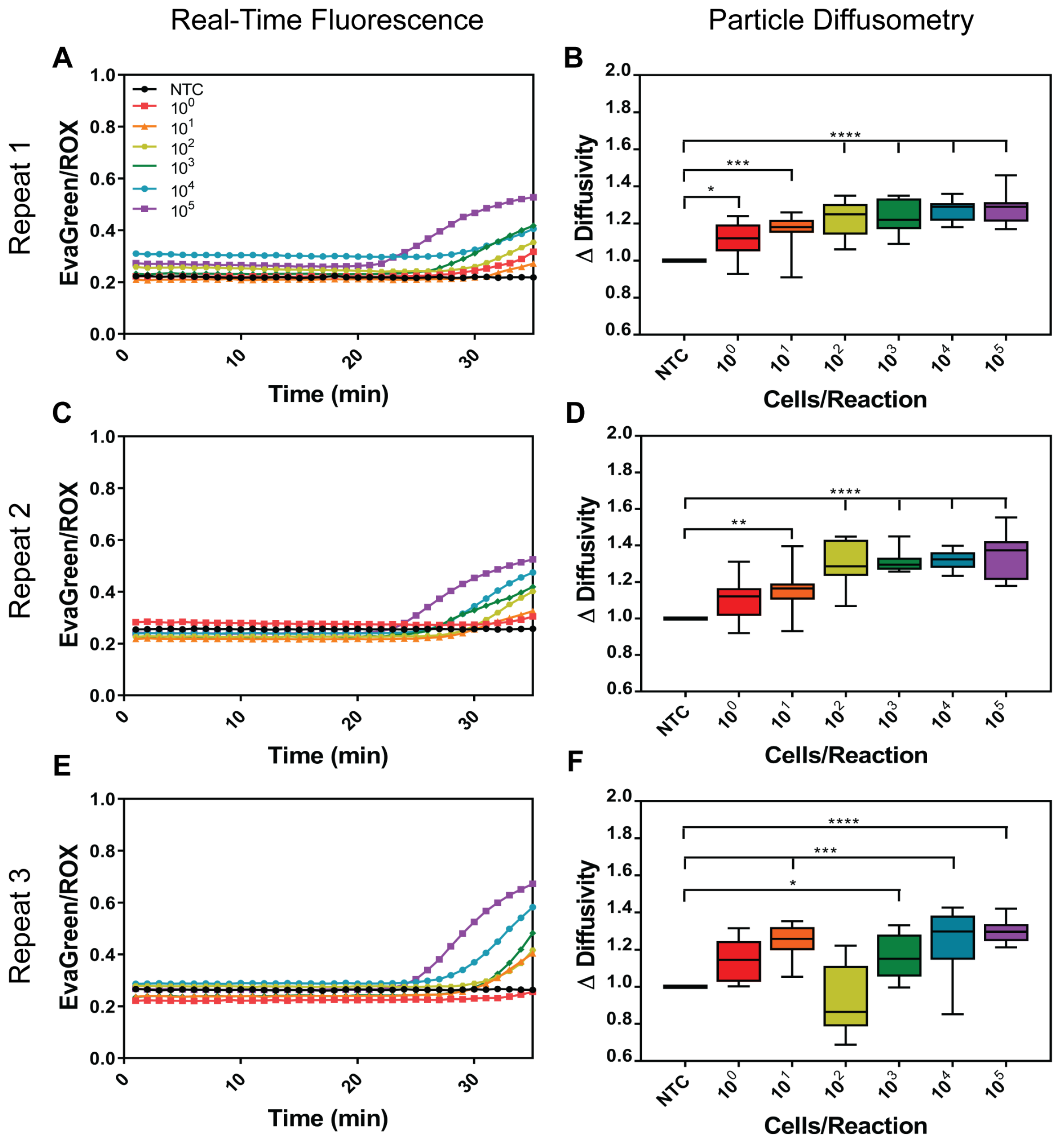

Fig. S6. Real-time fluorescence and PD measurements for biotinylated DNA from whole *V. cholerae* cell dilutions spiked in pond water. (A) Real-time fluorescent curves show amplification for different concentrations of cells for repeat 1. (B) PD measurements were performed on the same samples as in (A). PD measurements of diffusivity change for the 10^0, 10^1, 10^2, 10^3, 10^4, and 10^5 cells/reaction are statistically significantly different relative to NTC (* $p<0.05$, *** $p<0.001$, **** $p<0.0001$, **** $p<0.0001$, **** $p<0.0001$, **** $p<0.0001$, respectively). (C) Repeat 2 real-time fluorescent curves. (D) PD measurements were performed on the same samples as in (C). PD measurements of diffusivity change for the 10^1, 10^2, 10^3, 10^4, and 10^5 cells/reaction are statistically significantly different relative to NTC (** $p<0.01$, **** $p<0.0001$, **** $p<0.0001$, **** $p<0.0001$, **** $p<0.0001$, respectively). (E) Repeat 3 real-time fluorescent curves. (F) PD measurements were performed on the same samples as in (E). PD measurements of diffusivity change for the 10^1, 10^3, 10^4, and 10^5 cells/reaction are statistically significantly different relative to NTC (*** $p<0.001$, * $p<0.05$, *** $p<0.001$, **** $p<0.0001$, respectively). A one-way ANOVA post-hoc Dunnett's test was performed for all repeats with multiple comparisons against NTC with a 95% confidence interval.

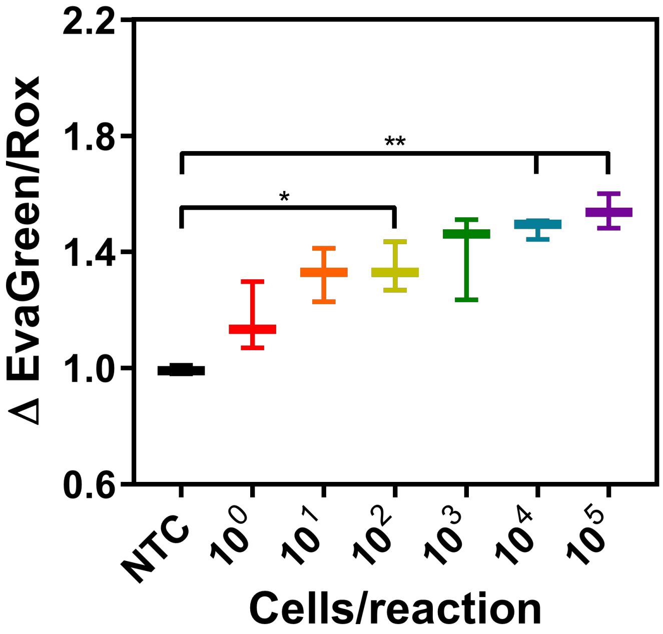

Fig. S7. Measuring LAMP amplification with fluorescence of *V. cholerae* whole cells in pond water. Cells were spiked into pond water at concentrations ranging from 10^0 – 10^5 cells/reaction. The change in EvaGreen/Rox is significant at 10^2 (* $p < 0.05$), 10^4, and 10^5 (** $p < 0.01$) cells/reaction. A one-way ANOVA post-hoc Dunnett's test was performed for all repeats with multiple comparisons against NTC with a 95% confidence interval.

Table S1. Nucleotide sequences of LAMP primers that target the *ctxA* gene.

Primer	Sequence (5' – 3')
B3	GTGGGCACTTCTCAAACT
F3	TCGGGCAGATTCTAGACC
BIP	TCAACCTTTATGATCATGCAAGAGGGGAAACATATCCATCATCGTG
FIP	TTGAGTACCTCGGTCAAAGTACTTCCTGATGAAATAAAGCAGTCA
LB	AACTCAGACGGGATTTGTTAGG
LF	CCTCTTGGCATAAGACCACC
Biotinylated LF	/5BiosG/CCTCTTGGCATAAGACCACC

Table S2. LAMP master mix used for amplification of *V. cholerae*.

**Reagent**	**Concentration**
Isothermal Buffer	1.0X
dNTPs	1.4 mM
Betaine	800 mM
F3 Primer	0.2 µM
B3 Primer	0.2 µM
FIP Primer	1.6 µM
BIP Primer	1.6 µM
LB Primer	1.6 µM
LF Primer	1.6 µM
EvaGreen Dye	0.4X
ROX Reference Dye	0.25 µL
Bst 2.0 Polymerase	8 U
Sample	2 µL
H2O	Fill to 25 µL

Table S3. Average data from blinded cholera study (n = 3). Particle diffusometry measurements were performed for four different groups. In the table (+) heat indicates the sample containing *V. cholerae* genomic DNA, (-) heat indicates the sample containing no *V. cholerae* genomic DNA which underwent the amplification process, (+) no heat is a sample containing *V. cholerae* genomic DNA that did not undergo amplification, and (-) no heat is a sample that does not contain *V. cholerae* genomic DNA and did not undergo heating.

**Sample**	(+) heat	(-) heat	(+) no heat	(-) no heat
**PD** (η/η_0)	1.25±0.01	1.00±0.02	1.02±0.01	1.07±0.01
